# PHY, MAC, and RLC Layer Based Estimation of Optimal Cyclic Prefix Length

**DOI:** 10.3390/s21144796

**Published:** 2021-07-14

**Authors:** Adriana Lipovac, Vlatko Lipovac, Borivoj Modlic

**Affiliations:** 1 Department of Electrical Engineering and Computing, University of Dubrovnik, 20000 Dubrovnik, Croatia; vlatko.lipovac@unidu.hr; 2 Faculty of Electrical Engineering and Computing, University of Zagreb, 10000 Zagreb, Croatia; borivoj.modlic@fer.hr

**Keywords:** cyclic prefix, OFDM, optimal length

## Abstract

This work is motivated by growing evidence that the standard Cyclic Prefix (CP) length, adopted in the Long Term Evolution (LTE) physical layer (PHY) specifications, is oversized in propagation environments ranging from indoor to typical urban. Although this ostensibly seems to be addressed by 5G New Radio (NR) numerology, its scalable CP length reduction is proportionally tracked by the OFDM symbol length, which preserves the relative CP overhead of LTE. Furthermore, some simple means to optimize fixed or introduce adaptive CP length arose from either simulations or models taking into account only the bit-oriented PHY transmission performance. On the contrary, in the novel crosslayer analytical model proposed here, the closed-form expression for the optimal CP length is derived such as to minimize the effective average codeblock length, by also considering the error recovery retransmissions through the layers above PHY—the Medium Access Control (MAC) and the Radio Link Control (RLC), in particular. It turns out that, for given protective coding, the optimal CP length is determined by the appropriate rms delay spread of the channel power delay profile part remaining outside the CP span. The optimal CP length values are found to be significantly lower than the corresponding industry-standard ones, which unveils the potential for improving the net throughput.

## 1. Introduction

The role of Cyclic Prefix (CP)—the copy of Orthogonal Frequency-Division Multiplexing (OFDM) symbol waveform tail inserted at the beginning is to mitigate multipath channel delay spreading and consequent intersymbol interference (ISI). Therefore, with appropriate time sampling of the received signal and the CP length at least slightly larger than the longest expected channel delay spread, not only the ISI but also the Intercarrier Interference (ICI) will be completely eliminated [1,2]. However, the price for that is paid in terms of spectral and power efficiency loss, as CP insertion reduces information throughput for the ratio of the CP length to the OFDM symbol period. This equals 7% for the so-called normal standard CP length of 4.69 μs, adopted in the Long Term Evolution (LTE) physical layer (PHY) specifications [3,4], wasting the transmitter energy, degrading the Signal-to-Noise Ratio (SNR), and shortening the mobile terminal battery life.

Still, there has been growing awareness that the fixed CP length of several times the rms delay spread, which state-of-the-art wireless systems use to accommodate to path length variations (of up to 1.4 km for LTE, in particular), is mostly oversized.

That is why, optimizing the fixed CP length [5,6,7] or implementing a channel-adaptive CP scheme [8,9] have been widely addressed by extensive simulations, providing various recommendations with this regard. However, only in few instances, a simple analytical approximation of the proclaimed optimal CP length being linear with the rms delay spread of the instantaneous power delay profile, was proposed [8], even adopting 2 as the universal linearity coefficient [10].

Moreover, CP length optimization ostensibly seems to be already addressed by the 5G New Radio (NR) numerology [11,12,13], as it allows scalable CP length reduction below the normal value of 4.69 μs. However, as it can be seen in Table 1, the proportional reduction of the OFDM symbol length, coming out of releasing the subcarrier spacing to increase above the (LTE’s) 15 kHz-only value, effectively preserves the constant CP overhead relative to the OFDM symbol duration [11,13]. This indicates that decreasing the standard CP length values through numerologies 1–4, provides no effective reduction of the CP overhead, as it would have for fixed subcarrier spacing.

Consequently, the LTE-centric CP length optimization model developed below remains equally applicable to 5G NR numerologies other than 0 (i.e., LTE), as well.

### 1.1. PHY-Only BER-Based Indication of Oversized CP Length

**Figure 1:**
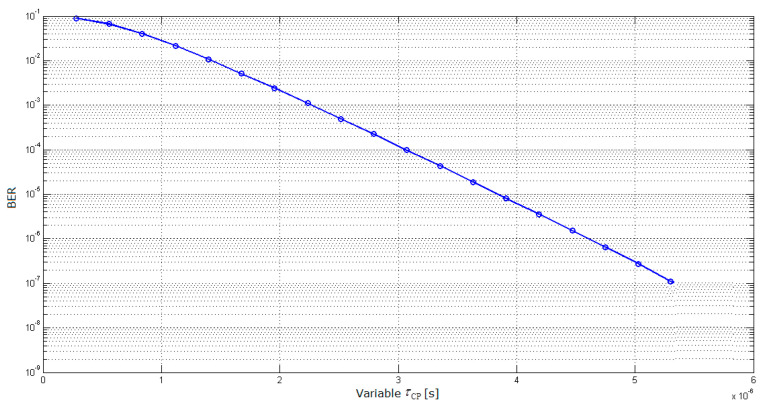
*BER* as a function of selected CP length (*τ*_CP_) [2].

In contrast to the other aforementioned investigations, mostly based on simulations [14,15,16,17,18], even using advanced channel models [19,20], the analytical expression for OFDM error floor (determined by time dispersion only) was derived allowing variable CP length [2]. This enabled testing appropriateness of the normal CP length by comparing it to the CP length values enabling the Bit Error Ratio (BER) values of 10^−3^ and 10^−6^ which are commonly referred to as the upper limits for degraded and acceptable bit-oriented digital transmission systems performance, respectively [21]. By applying these BER thresholds (in the absence of equivalent ones for the state-of-the-art access-level wireless networks of interest here), with long enough power delay profile, the corresponding CP length values were found to be around 2.25 and 4.60 μs, respectively, with very little BER reduction for CP length above 3 μs, Figure 1.

As it enables BER to take even lower value than the acceptability threshold aimed for quite demanding trunk-level transmission performance, the normal CP length of 4.69 μs is thus indicated to be unjustifiably oversized [2], unnecessarily overprotecting OFDM symbols against ISI at the expense of reduced throughput.

This indication motivates and justifies systematic reconsidering of the normal CP length optimality, specifically under conditions of low to moderate delay spreads, i.e., for propagation environments ranging from indoor to typical urban.

### 1.2. Motivation for PHY/MAC/RLC-Based CP Length Model

However, LTE and 5G NR specifications do not use BER at all as the PHY performance measure; rather, it is expressed in terms of Block Error Ratio (BLER), which determines the related BER and thus the corresponding CP length [22].

Moreover, in contrast to the what was elsewhere adopted, considering the PHY layer alone, in the following, we derive the optimal CP length based on the (overhead minimizing) compromise between the two mutually complementary data protection mechanisms: the CP itself at PHY layer and the block-oriented error protection by the Hybrid Automatic Repeat-reQuest (HARQ) error correction and detection/retransmission protocol spreading up through the Medium Access Control (MAC) layer, in conjunction with the Automatic Repeat-reQuest (ARQ) detection/retransmission protocol at the Radio Link Control (RLC) layer of the LTE protocol stack [22].

Consequently, as their coding gains partly take over the CP task of preventing errors due to ISI, it is reasonable to expect CP length that is shorter than the evidently oversized normal one to be sufficient to provide satisfactory protection but with less CP overhead.

In this regard, the inevitable question arises: what is the optimal redundancy trade-off between the CP length and the error recovery protocols that provides maximal throughput?

Therefore, in order to undoubtedly determine whether the standard CP length used in 4G and 5G networks is oversized (and if it is, to what extent), we need to develop an integrated crosslayer analytical model for CP length assessment and objectively determine its optimal value.

The organization of the paper is as it follows:

In Section 2, the expression for optimal CP length is derived by minimizing the Incremental-Redundancy HARQ (IR-HARQ) codeblock average gross length, which is for this purpose developed as a function of, sequentially, BLER, BER and CP length. Furthermore, in Section 3, the obtained analytical model is complemented at first by selecting the appropriate parameter values and then by computer simulation experiments. Conclusions are given in Section 4.

## 2. Optimal CP Length Model

It is well known that, in LTE systems, both IR-HARQ and ARQ functionalities are used. The former is run by both PHY and MAC layers; therefore, at the transmitter, during each transmit time interval (TTI), the transport block (TB) that PHY receives from MAC as “data” is extended by the 24-bits-long cyclic redundancy check (CRC) tail. This converts the TB into the codeblock, which is then turbo coded and rate-matched [22]. (Codeblock segmentation is not considered here, and the maximal codeblock length of 6144 bits is adopted, fitting in the maximal bandwidth of 5 MHz).

Up to four increasing-redundancy and rate-matched IR-HARQ redundancy versions (RV_0_ to RV_3_) of the codeblock can be sent until the codeblock CRC at the receiver indicates error-free transmission. Eventual residual post-HARQ erroneous codeblock is handed over to the RLC-layer ARQ process, which in that case makes the final retransmission [22].

### 2.1. Effective Average Codeblock Length

Let us consider HARQ and ARQ retransmissions of codeblocks at PHY/MAC and RLC layers, respectively, where we assume that the CRC error detection is always successful for all HARQ redundancy versions.

As we model retransmissions focusing on just the codeblock length, we accordingly abstract the HARQ and ARQ details with a simple and, in this case, adequate performance measure—the codeblock BLER.

Let us denote the nominal codeblock length as *L*_CB_ = *L* bits, and the BLER for the *i*-th redundancy version as: $BLER_{\mathrm{RV}i};i=0,1,2,3$. We can justifiably consider the latter as a monotonically decreasing function of CP length $\tau_{\mathrm{CP}}$; therefore, the larger $\tau_{\mathrm{CP}}$, the smaller $BLER_{\mathrm{RV}i}$. 

However, so far, there has been no analytical expression for $BLER_{\mathrm{RV}i}\left(\tau_{\mathrm{CP}}^{}\right)$, but we address this later.

Thus, the optimal CP length value is to be determined when the average gross count of transmitted bits—the effective average codeblock length:

(1) $$\begin{array}{ll} {\bar{L}}_{\mathrm{CB}}\left(\tau_{\mathrm{CP}}^{}\right) & =P\left(L\right)\cdot L+P\left(2L\right)\cdot 2L+P\left(3L\right)\cdot 3L+P\left(4L\right)\cdot 4L+P\left(5L\right)\cdot 5L\\ & =\left(1-BLER_{\mathrm{RV}0}\right)\cdot L+BLER_{\mathrm{RV}0}\cdot \left(1-BLER_{\mathrm{RV}1}\right)\cdot 2L+BLER_{\mathrm{RV}0}\cdot BLER_{\mathrm{RV}1}\cdot \left(1-BLER_{\mathrm{RV}2}\right)\cdot 3L\\ & +BLER_{\mathrm{RV}0}\cdot BLER_{\mathrm{RV}1}\cdot BLER_{\mathrm{RV}2}\cdot \left(1-BLER_{\mathrm{RV}3}\right)\cdot 4L+BLER_{\mathrm{RV}0}\cdot BLER_{\mathrm{RV}1}\cdot BLER_{\mathrm{RV}2}\cdot BLER_{\mathrm{RV}3}\cdot 5L\\ & =\left(1+\sum_{i=0}^{3}\prod_{j=0}^{i}BLER_{\mathrm{RV}j}\right)\cdot L \end{array}$$

which is needed for transmission of *L*-bits-long nominal codeblock (where *P*(*i*∙*L*); *i* = 1,2,3,4,5, denotes probability of transmitting a codeblock *i* times), is minimal.

With its first four terms, (1) reflects the IR-HARQ rule of transmitting another *L*-long RV*_i_* only if the just-finished transmission of RV*_i_*_−1_ results with CRC indication of an erroneous codeblock, where the probability of an error-free RV*_i_* is approximated by the 1-complement of the related *BLER*_RV*i*_. Thus, the overall used length equals *L* just for the error-free RV_0_, whereas it increases to 2*L* if the RV_0_ is erroneous, but RV_1_ is error-free. This rise continues to 3*L* if RV_0_ and RV_1_ are erroneous, but RV_2_ is error-free, and to 4*L* with erroneous RV_0_, RV_1_ and RV_2_, and error-free RV_3_ being the last IR-HARQ transmission. Finally, the overall length 5*L* is accumulated after erroneous RV_3_, as the post-HARQ remaining errors are dealt by RLC’s ARQ, which sends the last retransmission. Whether is it error-free (which is much more likely) or not is irrelevant for our CP length model, as no more retransmission is sent except, eventually, at the transmission layer, all the way up the stack, which is not in our scope here.

In this regard, a simple check of the sum of probabilities confirms that all mutually exclusive IR-HARQ and RLC-ARQ events (of having 1, 2, 3, 4 or 5 transmissions) are taken into account, i.e., that the sum of the coefficients in front of *L*, 2*L*, 3*L*, 4*L* and 5*L* in (1), equals unity. 

Apparently, ${\bar{L}}_{\mathrm{CB}}\left(\tau_{\mathrm{CP}}\right)$ is expressed in bits, whereas the CP is the extension of an OFDM symbol comprising $M_{\mathrm{OFDM}}=M$ modulation (pre-OFDM) symbols (as many as subcarriers), each representing $\mathrm{ld}M_{M-\mathrm{QAM}}=2,4,6$ bits, for 4, 16, and 64 QAM, respectively.

This means that each bit of an OFDM symbol carries the CP length fraction: $\frac{\tau_{\mathrm{CP}}}{M_{\mathrm{OFDM}}\cdot \mathrm{ld}M_{M-\mathrm{QAM}}}$, which implies that the duration of the CP-related part of ${\bar{L}}_{\mathrm{CB}}$ bits in (1) is:

(2) $${\bar{L}}_{\mathrm{CB}}\left(\tau_{\mathrm{CP}}^{}\right)\cdot \frac{\tau_{\mathrm{CP}}^{}}{\mathrm{ld}M_{M-\mathrm{QAM}}\cdot M_{\mathrm{OFDM}}}=L\cdot \left(1+\sum_{i=0}^{3}\prod_{j=0}^{i}BLER_{\mathrm{RV}j}\right)\cdot \frac{\tau_{\mathrm{CP}}^{}}{\mathrm{ld}M_{M-\mathrm{QAM}}\cdot M_{\mathrm{OFDM}}}$$

Minimizing (2) with respect to $\tau_{\mathrm{CP}}$ provides the optimal $\tau_{\mathrm{CP}}$ value:

(3) $$\frac{d}{d\tau_{\mathrm{CP}}^{}}\left[{\bar{L}}_{\mathrm{CB}}\left(\tau_{\mathrm{CP}}^{}\right)\cdot \tau_{\mathrm{CP}}^{}\right]=0$$

(We do not present here the cumbersome calculus of the second derivation to verify the extremum as minimum, as it is obvious from the concave graphs in Section 3).

Furthermore, before developing (3), let us express $BLER_{\mathrm{RV}i}\left(\tau_{\mathrm{CP}}^{}\right);i=1,2,3$, as it follows:

(4) $$BLER_{\mathrm{RV}i}\left(\tau_{\mathrm{CP}}^{}\right)=\frac{BLER_{\mathrm{RV}0}\left(\tau_{\mathrm{CP}}^{}\right)}{\Delta BLER_{\mathrm{RV}i/0}};i=1,2,3;\Delta BLER_{\mathrm{RV}i/0}>1$$

where we introduced the BLER reduction $\Delta BLER_{\mathrm{RV}i/0}>0$ of RV*_i_* with respect to the first transmission (RV_0_) at the same SNR value. (This is inverse to the definition of the more common coding gain $G_{\mathrm{RV}i/0}$, which allows RV*_i_* to have that much reduced SNR with respect to RV_0_ but still retain the same BLER value [22].)

Moreover, although CP length is determined exclusively by ISI (due to multipath propagation), we abstract it by an equivalent additive white-Gaussian noise (AWGN) source that would produce equal BLER degradation. In this way, it is possible to make use of the AWGN-based $\Delta BLER_{RVi}$ values that are already available for any particular selected value of the Channel Quality Indicator (CQI) [23,24].

**Figure 2:**
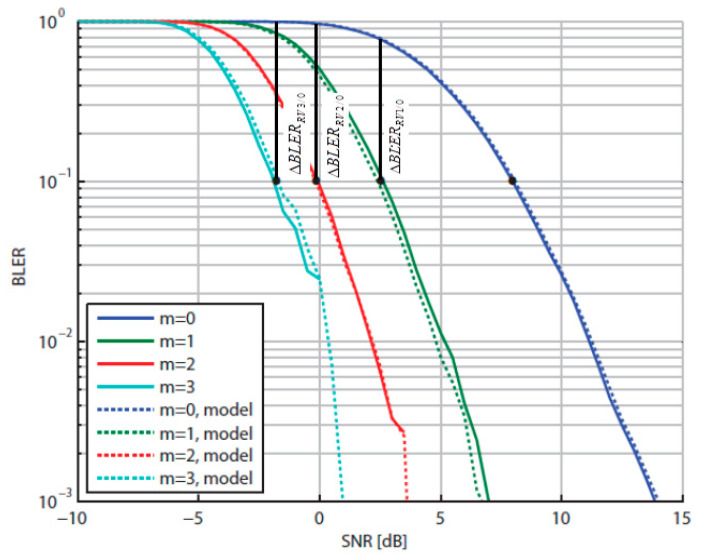
$BLER_{\mathrm{RV}m}=0.1;m=0,1,2,3$ vs. SNR for CQI = 6 [26]; added here: $\Delta BLER_{\mathrm{RV}m/0};m=1,2,3$.

Accordingly, in the exemplar Figure 2 for a small bandwidth, such as with 1 Physical Resource Block (PRB) conforming to 12 subcarriers [24,25], typical RV BLER curves (e.g., for CQI = 6) are shown [26], where the coding gains $G_{\mathrm{RV}i/0};i=1,2,3$, of an RV_*i*_ with respect to RV_0_, pertain to the target value of *BLER* = 0.1 [27,28].

According to (4), the successive BLER reductions between the RVs 1–3 and RV_0_ are represented in Figure 2 by the lengths of the vertical lines drawn from the points where the plots reach the target value $BLER_{\mathrm{RV}i}=10^{-1};i=1,2,3$ up to the intersections with the RV_0_ curve. Thus, in this example, we can see that $\Delta BLER_{\mathrm{RV}1/0}$, $\Delta BLER_{\mathrm{RV}2/0}$ and $\Delta BLER_{\mathrm{RV}3/0}$ are approximately equal to 7.5, 9.5 and 10 times, respectively. 

Furthermore, by substituting (4) into (2), the latter becomes:

(5) $${\bar{L}}_{\mathrm{CB}}\left(\tau_{\mathrm{CP}}\right)\cdot \tau_{\mathrm{CP}}=L\cdot \left(1+\sum_{i=1}^{4}\frac{BLER_{\mathrm{RV}0}^{i}}{\prod_{j=0}^{i-1}\Delta BLER_{\mathrm{RV}j/0}}\right)\cdot \tau_{\mathrm{CP}};\Delta BLER_{\mathrm{RV}0/0}=1$$

where we retained only $BLER_{\mathrm{RV}0}=BLER_{\mathrm{RV}0}\left(\tau_{\mathrm{CP}}^{}\right)$, which makes it easier to differentiate (5) with respect to $\tau_{\mathrm{CP}}$ in order to derive the optimal $\tau_{\mathrm{CP}}$ value.

(We justifiably consider that BLER reductions $\Delta BLER_{\mathrm{RV}i}$; *i* = 1,2,3, do not depend on $\tau_{\mathrm{CP}}$.)

### 2.2. Optimal CP Length for Minimal Codeblock Average Gross Length

Applying the general product differentiation rule to (5), from (3) we obtain:

(6) $$1+\sum_{i=1}^{4}\frac{BLER_{\mathrm{RV}0}^{i}\left(1+i\cdot \frac{\tau_{\mathrm{CP}}^{}}{BLER_{\mathrm{RV}0}}\cdot \frac{dBLER_{\mathrm{RV}0}}{d\tau_{\mathrm{CP}}^{}}\right)}{\prod_{j=0}^{i-1}\Delta BLER_{\mathrm{RV}j/0}}\approx 0;\Delta BLER_{\mathrm{RV}0/0}=1$$

As so far, there has been no evidence about a closed-form expression for $BLER_{\mathrm{RV}0}\left(\tau_{\mathrm{CP}}^{}\right)$ in (6), let us recall that its counterpart $BLER_{\mathrm{UNC}}$ for the uncoded blocks, can be expressed by its related *BER*_UNC_ [25]. By considering the time-dispersion (targeted by CP) dominant impairment causing errors, *BER*_UNC_ is a function of CP length: $BER_{\mathrm{UNC}}=BER\left(\tau_{\mathrm{CP}}^{}\right)$ [2].

If, analogously with the former inter-RV definition (4), we introduce $\Delta BLER_{\mathrm{RV}0/\mathrm{UNC}}$ as BLER reduction of the RV_0_ regarding the uncoded block transmission, then $BLER_{\mathrm{RV}0}\left(\tau_{\mathrm{CP}}^{}\right)$ can be expressed as:

(7) $$BLER_{\mathrm{RV}0}\left(\tau_{\mathrm{CP}}^{}\right)=\frac{BLER_{\mathrm{UNC}}\left[BER\left(\tau_{\mathrm{CP}}^{}\right)\right]}{\Delta BLER_{\mathrm{RV}0/\mathrm{UNC}}}$$

Thus, after simplifying the notation:

(8) $$BLER_{\mathrm{UNC}}\left[BER\left(\tau_{\mathrm{CP}}^{}\right)\right]=BLER$$

we express the derivation of $BLER_{\mathrm{RV}0}\left(\tau_{\mathrm{CP}}^{}\right)$ in (6) as follows:

(9) $$\frac{dBLER_{\mathrm{RV}0}\left(\tau_{\mathrm{CP}}^{}\right)}{d\tau_{\mathrm{CP}}^{}}=\frac{1}{\Delta BLER_{\mathrm{RV}0/\mathrm{UNC}}}\cdot \frac{d\left[BLER\left(BER\right)\right]}{dBER}\cdot \frac{dBER\left(\tau_{\mathrm{CP}}^{}\right)}{d\tau_{\mathrm{CP}}^{}}$$

To find the first left derivation on the right side of (9), we need to adopt a certain relationship between BLER and BER. In this regard, we can justifiably assume successful CP-aided mitigation of channel time dispersion, i.e., that the CP is long enough (e.g., as the normal one in LTE) to eliminate the vast majority of error bursts mostly arising from multipath propagation and retain only sporadic bit errors that mostly occur randomly and rarely in residual bursts (to be scattered by interleaving, anyway) [25].

However, although the common binomial distribution statistically well describes mutually independent bit error occurrences within an *L*-bits-long data block, in this case, we consider that the appropriate error generating model should still preserve (moderate) mutual dependability among the individual bit error occurrences. This conforms to the statistical model of sampling without replacement, well described by the hypergeometric distribution of errors within an errored data block (containing one or more erroneous bits), which provides the following *BLER* vs. *BER* relationship [25]:

(10) $$BLER\left(BER\right)\approx 1-{\left(1-BER\right)}^{L}$$

Thus, differentiating (10) leads to:

(11) $$\frac{d\left[BLER\left(BER\right)\right]}{dBER}=L\cdot {\left(1-BER\right)}^{L-1}$$

For large enough *L* (which is reasonable to presume), (11) can be rewritten as:

(12) $$\frac{d\left[BLER\left(BER\right)\right]}{dBER}\approx L\cdot {\left(1-BER\right)}^{L}$$

Now, we substitute $\frac{dBLER}{dBER}$ from (12) into (9) and then into (6) to make the latter related just to $BER=BER\left(\tau_{\mathrm{CP}}^{}\right)$:

(13) $$1+\sum_{i=1}^{4}\frac{\frac{{\left[1-{\left(1-BER\right)}^{L}\right]}^{i}}{\Delta BLER_{\mathrm{RV}0/\mathrm{UNC}}}\left(1+i\cdot \frac{{\left(1-BER\right)}^{L}}{1-{\left(1-BER\right)}^{L}}\cdot L\cdot \tau_{\mathrm{CP}}^{}\cdot \frac{dBER}{d\tau_{\mathrm{CP}}^{}}\right)}{\prod_{j=0}^{i-1}\Delta BLER_{\mathrm{RV}j/0}}\approx 0;\Delta BLER_{\mathrm{RV}0/0}=1$$

Let us point out here that in the following, we simplified some expressions to ease their interpretation, by applying several analytically justifiable approximations that we also double-checked step-by-step with MATLAB tool and kept only the ones with negligible impact on the final results.

Furthermore, as we target the propagation environments ranging from indoor to typical urban [22], even the latter one’s 3GPP-assigned median rms delay spread of 500 ns is nowadays practically reduced down to just about 200 ns (due to near antennas, mounted to walls and building rooftops). Thus, the CP of a couple of microseconds spanning the “lion’s part” of the power delay profile might leave just extremely rare long delay excursions out of its reach to eventually produce accordingly rare bit errors, represented by very small BER values.

Therefore, for long enough CP, i.e., with effective CP-aided protection against ISI, we may always consider that *BER* << 1. Indeed, even adopting the 3GPP-targeted PHY/MAC transmission performance value of *BLER* = 10^−1^ [22] and the maximal block length of *L* = 6144 bits in (10) results in a small value of *BER* ≈ 1.63∙10^−5^.

This implies that we can also justifiably assume that: L·BER<<1, and, consequently, can take into account only the first two terms in the binomial expansion:

(14) $${\left(1-BER\right)}^{L}=\sum_{i=0}^{L}\left(\begin{array}{c} L\\ i \end{array}\right)\cdot 1^{L-i}\cdot {\left(-BER\right)}^{i}\approx 1-L\cdot BER$$

Furthermore, by applying (14) into (13) and associating the terms with equal powers, (13) can be rewritten in the more concise form as it follows:

(15) $$1+\sum_{i=1}^{4}\frac{{\left(L\cdot BER\right)}^{i}\cdot \left(1+i\cdot \frac{1-L\cdot BER}{BER}\cdot \tau_{\mathrm{CP}}^{}\cdot \frac{dBER}{d\tau_{\mathrm{CP}}^{}}\right)}{\Delta BLER_{\mathrm{RV}0/\mathrm{UNC}}\cdot \prod_{j=0}^{i-1}\Delta BLER_{\mathrm{RV}j/0}}\approx 0;\Delta BLER_{\mathrm{RV}0/0}=1$$

To solve (15) with respect to $\tau_{\mathrm{CP}}$ and thus find the optimal CP length, we need to know $BER\left(\tau_{\mathrm{CP}}^{}\right)$ and then derive $\frac{dBER\left(\tau_{\mathrm{CP}}^{}\right)}{d\tau_{\mathrm{CP}}^{}}$, too.

### 2.3. Time-Dispersion-Only Related Residual BER for Optimal CP Length

As the CP mechanism is aimed to mitigate exclusively the channel time dispersion (i.e., multipath propagation causing ISI and error bursts), the standard CP length values—the normal and the extended one [22], assigned to urban or rural environment, respectively, so were determined exclusively by power delay profile’s delay spread, without any regard to noise.

Accordingly, for the purpose of assessing the overhead of the actual standard CP length, we stick to its framework of ISI-dominant conditions, by not considering noise even in propagation environments exceeding indoor or small-cell dimensions (where this assumption mostly holds anyway). 

However, in contrast to the above-reviewed PHY-only-based standard CP length(s) chosen to be slightly larger than the expected maximal delay spread of the channel power delay profile, taking into account HARQ/RLC-ARQ retransmissions as well, reveals that, for any chosen fixed CP length—be it the industry-standard normal one or any other—any noise level would increase the rate of retransmissions and so reduce the effective throughput.

Consequently, taking into account noise in the CP length optimization would point to even smaller optimal value, which would further imply the industry-standard normal CP length to be considered even more oversized. This is quite unrealistic and would finally end up in a reductio ad absurdum situation when noise becomes dominant impairment, causing almost all HARQ/RLC-ARQ retransmissions and making the fixed pre-assigned CP to become just the burden wasting the bandwidth and energy. 

Therefore, including noise into the model would make sense only if we introduce a sort of noise-adaptive (i.e., CQI/MCS adaptive) CP length, in a way analogous to the time-dispersion-adaptive schemes mentioned in the introduction.

However, this is not what we are proposing here, as it would have not been compatible with 4G/5G standards and would therefore be difficult to implement and get accepted by the industry. 

Moreover, as reducing CP length effectively increases the energy per symbol, i.e., SNR, this makes retransmissions less frequent and partly compensates for the noise effect.

Therefore, the residual BER being determined just by the ISI—the OFDM error floor—is to be adopted here.

With this regard, we consider the utmost general, quasistatic wide-sense stationary uncorrelated scattering (WSSUS) multipath channel model, whose impulse response is the sum of *N* complex delta functions with powers $A_{i}^{2}$, phases $\psi_{i}$ and delays $\tau_{i}$, i=1,2,…N [2]. Consequently, the received signal is the sum of accordingly scaled, phase-shifted and delayed replicas—echoes of the transmitted signal—where selecting any sampling instant (that is closest to $N_{}^{-}$-th out of *N* impulses of the profile), distinguishes the preceding (“−”) echoes from the delayed (“+”) ones with their accordingly denoted “−” and “+” powers, phases and delays.

The *m*-th modulation (pre-OFDM) symbol $s_{m,n}e^{j\phi_{m,n}}$, with period $T_{s}$, is aggregated with other ones into the actual *n*-th transmitted OFDM symbol: $\sum_{m=1}^{M}s_{m,n}e^{j\phi_{m,n}}\cdot e^{jm\cdot \frac{2\pi }{MT_{s}}\tau }$, where *M* is the number of subcarriers and τ is the delay with respect to the sampling instant. Analogously, the (*n* − 1)-th and the (*n* + 1)-th transmitted OFDM symbols in the observed data sequence, are represented as $\sum_{m=1}^{M}s_{m,n-1}e^{j\phi_{m,n-1}}\cdot e^{jm\cdot \frac{2\pi }{MT_{s}}\tau }$ and $\sum_{m=1}^{M}s_{m,n+1}e^{j\phi_{m,n+1}}\cdot e^{jm\cdot \frac{2\pi }{MT_{s}}\tau }$, respectively, [2].

Then, for given channel and signal statistical parameters, the error floor prediction is [2]:

(16) $$\begin{array}{l} BER_{BPSK}=\frac{1}{2\sqrt{\pi }}(\sqrt{W^{-}E\left[{\left(\frac{\tau_{i}^{-}}{T_{s}}\right)}^{2}\right]\cdot \mathrm{var}\left[\Delta s_{n/n+1}^{}\right]}+\sqrt{W^{+}E\left[{\left(\frac{\tau_{i}^{+}}{T_{s}}\right)}^{2}\right]\cdot \mathrm{var}\left[\Delta s_{n-1/n}^{}\right]}\\ -\frac{\sqrt{W^{-}E\left[{\left(\frac{\tau_{i}^{-}}{T_{s}}\right)}^{2}\right]\cdot \mathrm{var}\left[\Delta s_{n/n+1}^{}\right]}\cdot \sqrt{W^{+}E\left[{\left(\frac{\tau_{i}^{+}}{T_{s}}\right)}^{2}\right]\cdot \mathrm{var}\left[\Delta s_{n-1/n}^{}\right]}}{\sqrt{W^{-}E\left[{\left(\frac{\tau_{i}^{-}}{T_{s}}\right)}^{2}\right]\cdot \mathrm{var}\left[\Delta s_{n/n+1}^{}\right]}+\sqrt{W^{+}E\left[{\left(\frac{\tau_{i}^{+}}{T_{s}}\right)}^{2}\right]\cdot \mathrm{var}\left[\Delta s_{n-1/n}^{}\right]}}) \end{array}$$

where “−” and “+” rms delay spreads:

(17) $$\sqrt{E\left[{\left(\frac{\tau_{i}^{-}}{T_{s}}\right)}^{2}\right]}=\sqrt{\frac{\sum_{i=1}^{N^{-}}{\left(A_{i}^{-}\right)}^{2}{\left(\frac{\tau_{i}^{-}}{T_{s}}\right)}^{2}}{\sum_{i=1}^{N^{-}}{\left(A_{i}^{-}\right)}^{2}}};\sqrt{E\left[{\left(\frac{\tau_{i}^{+}}{T_{s}}\right)}^{2}\right]}=\sqrt{\frac{\sum_{i=N^{-}+1}^{N}{\left(A_{i}^{+}\right)}^{2}{\left(\frac{\tau_{i}^{+}}{T_{s}}\right)}^{2}}{\sum_{i=1}^{N^{-}}{\left(A_{i}^{+}\right)}^{2}}}$$

of the power delay profile, are weighted by their corresponding “−” and “+” aggregate powers:

(18) $$W^{-}=\sum_{i=1}^{N^{-}}{\left(A_{i}^{-}\right)}^{2};W^{+}=\sum_{i=N^{-}+1}^{N}{\left(A_{i}^{+}\right)}^{2};W^{-}+W^{+}=\sum_{i=1}^{N}A_{i}^{2}=1$$

respectively, whereas the variances of differences between the actual (*n*-th) OFDM symbol and the following one, as well as between the preceding OFDM symbol and the actual one:

(19) $$\mathrm{var}\left[\Delta s_{n/n+1}^{}\right]=\sum_{m=1}^{M}\frac{s_{m,n}^{2}+s_{m,n+1}^{2}}{M^{2}},\mathrm{var}\left[\Delta s_{n-1/n}^{}\right]=\sum_{m=1}^{M}\frac{s_{m,n-1}^{2}+s_{m,n}^{2}}{M^{2}}$$

respectively, are normalized to the modulation symbol.

From (17)–(19), and finally from (16), it is obvious that: $BER=BER\left(\tau_{\mathrm{CP}}^{}\right)$, as the effect of CP is modeled simply by discarding the power-delay profile terms having delays within $\tau_{\mathrm{CP}}$ span around the sampling instant, i.e., having indices in the range from $N_{}^{-}-N\left(\tau_{\mathrm{CP}}^{-}\right)$ to $N\left(\tau_{\mathrm{CP}}^{+}\right)\text{>}N_{}^{-}$, where $N\left(\tau_{\mathrm{CP}}^{-}\right)$ and $N\left(\tau_{\mathrm{CP}}^{+}\right)$ are the according “−” and “+” CP spans, respectively.

Moreover, as the BER expression (16) does not presume any specific sampling instant, for any power delay profile with dominant first path power ($A_{1}^{2}$), the optimal sampling was found to provide just minor BER difference regarding sampling at the mean delay or just upon the first arrival [1].

Consequently, when the latter is adopted, the “−” terms in (16) can be discarded, whereas the remaining “+” ones could be written without the superscript; therefore, (16) simplifies to:

(20) $$BER\left(\tau_{\mathrm{CP}}^{}\right)=\frac{1}{2\sqrt{\pi }\cdot T_{s}}\sqrt{E\left[\Delta s_{n-1/n}^{2}\right]}\cdot \sqrt{\sum_{i=N\left(\tau_{\mathrm{CP}+}^{}\right)}^{N}A_{i}^{2}\tau_{i}^{2}}$$

where “+” in “CP+” indicates that the first term in the sum is with delay just slightly above $\tau_{\mathrm{CP}}$.

Specifically, for BPSK modulation, as $s_{m,n}$ and $s_{m,n+1}$ become bipolar, taking the values ±1 each, (19) transforms to:

(21) $$\begin{array}{ll} E\left[\Delta s_{n/n+1}^{2}\right] & =E\left[\Delta s_{n-1/n}^{2}\right]\\ & =\sum_{m=1}^{M}\frac{s_{m,n}^{2}+s_{m,n+1}^{2}}{M^{2}}=\frac{M\cdot \left[{\left(\frac{1}{\sqrt{2}}\right)}^{2}+{\left(\frac{1}{\sqrt{2}}\right)}^{2}\right]}{M^{2}}=\frac{1}{M} \end{array}$$

so that BER further simplifies from (20) to:

(22) $$BER\left(\tau_{\mathrm{CP}}^{}\right)=\frac{1}{2\sqrt{\pi \cdot M}}\cdot \frac{\sqrt{\sum_{i=N\left(\tau_{CP}^{}+\right)}^{N}A_{i}^{2}\tau_{i}^{2}}}{T_{s}}=\frac{1}{2\sqrt{\pi \cdot M}}\cdot \frac{\sqrt{\overset{-}{\tau_{\mathrm{CP}+}^{2}}}}{T_{s}}$$

where $\sqrt{\overset{-}{\tau_{\mathrm{CP}+}^{2}}}=\sqrt{\sum_{i=N\left(\tau_{\mathrm{CP}+}^{}\right)}^{N}A_{i}^{2}\tau_{i}^{2}}$ is the “CP-residual” (i.e., after the “cut-off” $\tau_{\mathrm{CP}}$) rms delay spread, which therefore monotonically decreases with $\tau_{\mathrm{CP}}$.

Let us make an observation here that, for large enough (i.e., effective) $\tau_{\mathrm{CP}}$, the residual (“high-passed”) delay profile weighting coefficients $A_{i}^{2}$ are very small, and so is $\sqrt{\overset{-}{\tau_{\mathrm{CP}+}^{2}}}$ with respect to $2\sqrt{\pi \cdot M}\cdot T_{s}$. The consequently very small BER values justify the approximation (14) that is finally confirmed in Section 3. 

Furthermore, for the higher-order modulations applied in LTE, namely: 4 QAM, 16 QAM and 64 QAM with Gray constellation mapping, the coefficient $k_{\mathrm{MOD}}$ should be inserted in (22) [2]:

(23) $$BER\left(\tau_{\mathrm{CP}}^{}\right)=\frac{k_{\mathrm{MOD}}}{2\sqrt{\pi \cdot M}}\cdot \frac{\sqrt{\sum_{i=N\left(\tau_{CP+}^{}\right)}^{N}A_{i}^{2}\tau_{i}^{2}}}{T_{s}}=\frac{k_{\mathrm{MOD}}}{2\sqrt{\pi \cdot M}}\cdot \frac{\sqrt{\overset{-}{\tau_{\mathrm{CP}+}^{2}}}}{T_{s}}$$

where:

(24) $$k_{\mathrm{MOD}}=\frac{\frac{SER_{M\mathrm{QAM}}}{BER_{\mathrm{BPSK}}}}{ldM}=\{\begin{array}{c} 2/2=1;4\mathrm{QAM}\\ 3/4=0.75;16\mathrm{QAM}\\ 3.5/6=0.583;64\mathrm{QAM} \end{array}$$

Now, we differentiate (23):

(25) $$\frac{dBER\left(\tau_{\mathrm{CP}}^{}\right)}{d\tau_{\mathrm{CP}}^{}}=\frac{k_{\mathrm{MOD}}}{2\sqrt{\pi \cdot M}\cdot T_{s}}\cdot \frac{d\left[\sqrt{\sum_{i=N\left(\tau_{CP+}^{}\right)}^{N}A_{i}^{2}\tau_{i}^{2}}\right]}{d\tau_{\mathrm{CP}}^{}}$$

where:

(26) $$\frac{d\left[\sqrt{\sum_{i=N\left(\tau_{CP+}^{}\right)}^{N}A_{i}^{2}\tau_{i}^{2}}\right]}{d\tau_{\mathrm{CP}}^{}}=\frac{1}{2}\frac{1}{\sqrt{\sum_{i=N\left(\tau_{CP+}^{}\right)}^{N}A_{i}^{2}\tau_{i}^{2}}}\cdot \frac{d\left[\sum_{i=N\left(\tau_{CP+}^{}\right)}^{N}A_{i}^{2}\tau_{i}^{2}\right]}{d\tau_{\mathrm{CP}}^{}}$$

and:

(27) $$\begin{array}{ll} \frac{d\left[\sum_{i=N\left(\tau_{CP+}^{}\right)}^{N}A_{i}^{2}\tau_{i}^{2}\right]}{d\tau_{\mathrm{CP}}^{}} & =\underset{\Delta \tau_{\mathrm{CP}}^{}\to 0}{\lim}\frac{\sum_{i=N\left(\tau_{CP+}^{}+\Delta \tau_{CP}^{}\right)}^{N}A_{i}^{2}\tau_{i}^{2}-\sum_{i=N\left(\tau_{CP+}^{}\right)}^{N}A_{i}^{2}\tau_{i}^{2}}{\Delta \tau_{\mathrm{CP}}^{}}\\ & \approx \underset{\Delta \tau_{\mathrm{CP}}^{}\to \Delta \tau }{\lim}\frac{-\sum_{i=N\left(\tau_{CP}^{}\right)}^{N\left(\tau_{CP+}^{}+\Delta \tau_{CP}^{}\right)}A_{i}^{2}\tau_{i}^{2}}{\Delta \tau_{\mathrm{CP}}^{}}=-\frac{A_{N\left(\tau_{\mathrm{CP}+}^{}\right)}^{2}}{\Delta \tau }\cdot \tau_{\mathrm{CP}}^{2};\\ & \;\Delta \tau \leq \Delta \tau_{\mathrm{CP}}^{} \end{array}$$

where Δτ is the minimal measurable delay interval—discrete quantum of the power delay profile.

Substituting (27) into (26) and then (26) into (25), we obtain:

(28) $$\begin{array}{ll} \frac{dBER\left(\tau_{\mathrm{CP}}^{}\right)}{d\tau_{\mathrm{CP}}^{}} & =-\frac{k_{\mathrm{MOD}}}{4\sqrt{\pi \cdot M}\cdot T_{s}}\cdot \frac{A_{N\left(\tau_{\mathrm{CP}+}^{}\right)}^{2}}{\Delta \tau \cdot \sqrt{\sum_{i=N\left(\tau_{CP+}^{}\right)}^{N}A_{i}^{2}\tau_{i}^{2}}}\cdot \tau_{\mathrm{CP}}^{2}\\ & =-\frac{k_{\mathrm{MOD}}}{4\sqrt{\pi \cdot M}\cdot T_{s}}\cdot \frac{A_{N\left(\tau_{\mathrm{CP}+}^{}\right)}^{2}}{\Delta \tau \cdot \sqrt{\overset{-}{\tau_{\mathrm{CP}+}^{2}}}}\cdot \tau_{\mathrm{CP}}^{2} \end{array}$$

Finally, we substitute BER from (23) and $\frac{dBER\left(\tau_{\mathrm{CP}}^{}\right)}{d\tau_{\mathrm{CP}}^{}}$ from (28) into (15), so the latter becomes:

(29) $$1+\sum_{i=1}^{4}\frac{{\left(\frac{L\cdot k_{\mathrm{MOD}}}{2\sqrt{\pi \cdot M}}\cdot \frac{\sqrt{\overset{-}{\tau_{\mathrm{CP}+}^{2}}}}{T_{s}}\right)}^{i}}{\Delta BLER_{\mathrm{RV}0/\mathrm{UNC}}\cdot \prod_{j=0}^{i-1}\Delta BLER_{\mathrm{RV}j/0}}\cdot \left[1-\frac{i}{2}\cdot \left(1-\frac{L\cdot k_{\mathrm{MOD}}}{2\sqrt{\pi \cdot M}}\cdot \frac{\sqrt{\overset{-}{\tau_{\mathrm{CP}+}^{2}}}}{T_{s}}\right)\cdot \frac{A_{N\left(\tau_{\mathrm{CP}+}^{}\right)}^{2}}{\overset{-}{\tau_{\mathrm{CP}+}^{2}}\cdot \Delta \tau }\cdot \tau_{\mathrm{CP}}^{3}\right]=0;\Delta BLER_{\mathrm{RV}0/0}=1$$

The trade-off between the CP length $\tau_{\mathrm{CP}}$ and the BLER reductions $\Delta BLER_{\mathrm{RV}i/0}$ (and, consequently, the related HARQ coding gains) is evident in (29), as each addend of the sum in (29) monotonically decreases either by increasing $\tau_{\mathrm{CP}}$ (i.e., by decreasing the normalized “CP-residual” rms delay spread $\sqrt{\overset{-}{\tau_{\mathrm{CP}+}^{2}}}$), or by increasing the BLER reductions in the denominator (Analogous CP–HARQ trade-off is evident with decreasing $\tau_{\mathrm{CP}}$). 

Thus, (29) expresses the $\tau_{\mathrm{CP}}$-optimizing “balance” between the two protecting mechanisms enabling minimal average codeblock gross length. 

This implies that selecting the 3GPP-LTE modulation and coding scheme (MCS), i.e., the channel quality identifier (CQI) with certain $\Delta BLER_{\mathrm{RV}i/0}$, determines the achievable $\tau_{\mathrm{CP}}$ reduction and vice versa; therefore, there must be an optimal CP length that enables maximal overall throughput.

Moreover, (29) can be significantly simplified without much accuracy degradation, by neglecting the two higher-order addends (due to their fast increasing denominators and decreasing numerators). This reflects the well-known IR-HARQ feature that most of the coding gain is with the first round(s) [22].

Likewise, the expression (5) for the CP-related duration ${\bar{L}}_{\mathrm{CB}}\left(\tau_{\mathrm{CP}}\right)\cdot \tau_{\mathrm{CP}}$ of average gross data ${\bar{L}}_{\mathrm{CB}}$ bits transmitted for a codeblock is developed by substituting (10), (14) and (23) in (5), which results in:

(30) $${\bar{L}}_{\mathrm{CB}}\left(\tau_{\mathrm{CP}}\right)\cdot \tau_{\mathrm{CP}}=L\cdot \left[1+\sum_{i=1}^{4}\frac{{\left(\frac{L\cdot k_{\mathrm{MOD}}}{2\sqrt{\pi \cdot M}}\cdot \frac{\sqrt{\overset{-}{\tau_{\mathrm{CP}+}^{2}}}}{T_{s}}\right)}^{i}}{\Delta BLER_{\mathrm{RV}0/\mathrm{UNC}}\cdot \prod_{j=0}^{i-1}\Delta BLER_{\mathrm{RV}j/0}}\right]\cdot \tau_{\mathrm{CP}};\Delta BLER_{\mathrm{RV}0/0}=1$$

As $\tau_{\mathrm{CP}}$ increases, the “CP-residual” rms delay spread $\sqrt{\overset{-}{\tau_{\mathrm{CP}+}^{2}}}$ and thus the whole expression in the squared brackets of (30), monotonically decreases, thus opposing the $\tau_{\mathrm{CP}}$ rise and paving the way to existence of minimal ${\bar{L}}_{\mathrm{CB}}\left(\tau_{\mathrm{CP}}\right)\cdot \tau_{\mathrm{CP}}$.

Finally, let us note once again that all approximations in this section were step-by-step verified by computer simulations.

## 3. Numerical Results

### 3.1. Setup of Coding and Channel Parameters

#### 3.1.1. Power-Delay Profile

Actually, (29) and (30) are valid for any power delay profile, but their numerical verification requires adopting a certain power delay profile shape (that is not uniquely determined just by the rms delay spread value).

However, as the standard wireless channel models (ITU, 3GPP) are delay limited and with poor delay resolution, these are not appropriate for the CP length testing in propagation environments ranging from indoor to typical urban. In this regard, a convenient solution is to mimic the unlimited power delay profiles by the limited exponential profile in particular, where for any rms delay spread of interest here (100, 200, 300 or 400 ns), we can design how long is the profile (i.e., its maximal delay) by a priori choosing the probability *p* that all impulses of the corresponding delay-unlimited exponential profile of equal rms delay spread are within the selected maximal delay [2]. Thus, e.g., for *p* = 99%, the maximal delay is 4.6 times the rms delay spread, whereas for *p* = 99.9999999999999%, this factor equals 34.5. This way, the impact of CP length can be tracked with as fine delay resolution as needed. The minimal measurable delay interval in (29)—discrete quantum of the power delay profile—is chosen to take: $\Delta \tau =5\cdot 10^{-7}$ seconds.

#### 3.1.2. BLER Reductions

Now, let us consider which values to adopt for the IR-HARQ BLER reductions $\Delta BLER_{\mathrm{RV}i/0};i=1,2,3$. 

As the goal here is to find the optimal value for the evidently oversized standard CP length(s), it is appropriate to adopt such values of the relevant parameters that will not themselves contribute to CP length shortening but oppose it. Accordingly, as it is already pointed out regarding (29), the minimal BLER reduction and so the coding gain alike, i.e., the lowest-order MCS/CQI is the best choice in this regard [26].

However, graphical means presented in the exemplar Figure 2 as applicable for a small bandwidth is of no use for wider bandwidth, such as with typically 25 PRBs, which also implies larger codeblock size (of interest here). 

**Figure 3:**
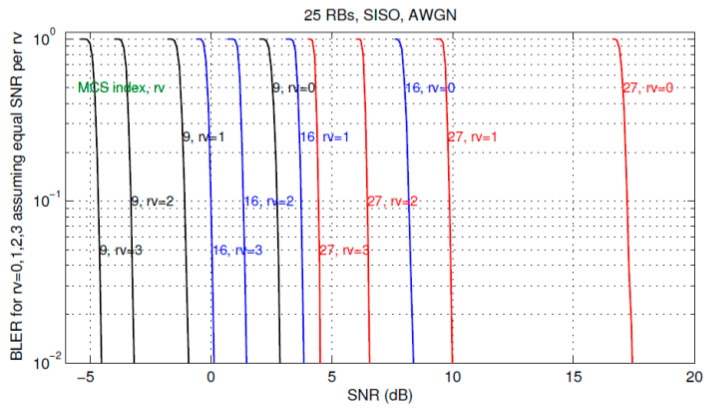
AWGN $BLER_{\mathrm{RV}i};i=0,1,2,3$ for various RVs, for MCS = 9, 16, 27 [23].

Then, the *BLER*(*SNR*) curves are getting very steep—almost vertical—taking on a waterfall shape, as it can be seen in the exemplar Figure 3 [23].

Apparently, each RV BLER curve is mostly in its “saturation” state (when all blocks are erroneous, i.e., *BLER* = 1) before entering its waterfall segment to start plunging down to the referential *BLER* = 10^−1^ level. Applying the same graphical means to measure *BLER* reduction as in Figure 2, by drawing vertical lines from the *BLER* = 10^−1^ points up to the RV_0_ curve, it is obvious that all BLER reductions are almost equal to 1/0.1, i.e., $\Delta BLER_{\mathrm{RV}i/0}=10,i=1,2,3$.

Thus, for large bandwidth, there are no specific worst-case-scenario IR-HARQ parameters (with smallest BLER reductions) to be selected for the optimal CP length model.

This applies for somewhat smaller bandwidths as well, e.g., for 5 MHz, when large codeblock lengths are still used (even though not necessarily the maximal one of 6144 bits allowed by the turbo coder). Whatever the case, the larger the codeblock, the steeper the BLER curves [23].

Now, let us analytically verify that these $\Delta BLER_{\mathrm{RV}i/0}$ values are generally applicable in the proposed CP optimization model.

Thus, having chosen a certain MCI/CQI value (and so the modulation type and the coding gains $G_{\mathrm{RV}i/0};i=1,2,3$), we can model $\Delta BLER_{\mathrm{RV}i/0}$ simply by focusing on the actual $BER_{\mathrm{RV}0}$ (rather than $BLER_{\mathrm{RV}0}$), in order to apply $G_{\mathrm{RV}i/0}$ to the classic BER expression for the AWGN channel [25]:

(31) $$BER_{\mathrm{RV}0}=k_{M\mathrm{QAM}}\cdot Q\left(\sqrt{2\cdot {\left(\frac{E_{b}}{N_{0}}\right)}_{\mathrm{RV}0}}\right)$$

In this regard, let us first develop $\Delta BLER_{\mathrm{RV}i/0}$ from (4):

(32) $$\Delta BLER_{\mathrm{RV}i/0}=\frac{BLER_{\mathrm{RV}0}\left(\tau_{\mathrm{CP}}^{}\right)}{BLER_{\mathrm{RV}i}\left(\tau_{\mathrm{CP}}^{}\right)};i=1,2,3\Delta BLER_{\mathrm{RV}i/0}>1$$

as it follows:

(33) $$\Delta BLER_{\mathrm{RVi}/0}=\frac{BLER_{\mathrm{RV}0}\left[BER_{\mathrm{RV}0}\left(\frac{\frac{E_{b}}{N_{0}}}{G_{\mathrm{RV}i/0}}\right)\right]}{BLER_{\mathrm{RV}0}\left[BER_{\mathrm{RV}0}\left(\frac{E_{b}}{N_{0}}\right)\right]};i=1,2,3$$

where $G_{\mathrm{RV}i/0}$ is not considered here as the coding gain that enables the higher-order RV*_i_* to preserve the BLER of RV_0_ with $G_{\mathrm{RV}i/0}$ times lower energy per bit to noise power spectral density ratio *E*_b_/*N*_0_ but as the increase in the RV_0′s_ *E*_b_/*N*_0_ that makes its $BLER_{\mathrm{RV}0}$ reduced to $BLER_{\mathrm{RVi}}$. 

Taking into account (10), (33) is expressed as:

(34) $$\Delta BLER_{\mathrm{RVi}/0}=\frac{1-{\left[1-BER_{\mathrm{RV}0}\left(\frac{\frac{E_{b}}{N_{0}}}{G_{\mathrm{RV}i/0}}\right)\right]}^{L}}{1-{\left[1-BER_{\mathrm{RV}0}\left(\frac{E_{b}}{N_{0}}\right)\right]}^{L}};i=1,2,3$$

Furthermore, applying (14) into (34) leads to:

(35) $$\Delta BLER_{\mathrm{RVi}/0}\approx \frac{BER_{\mathrm{RV}0}\left(\frac{\frac{E_{b}}{N_{0}}}{G_{\mathrm{RVi}/0}}\right)}{BER_{\mathrm{RV}0}\left(\frac{E_{b}}{N_{0}}\right)}=\Delta BER_{\mathrm{RVi}/0};i=1,2,3$$

Thus, according to (35), the HARQ-made BLER reductions are now expressed by the according BER reductions and so can be easily estimated by applying (31) to RV_0_, with and without the coding gain $G_{\mathrm{RV}i/0}$.

Generally, according to (14), the target *BLER* = 10^−1^ is achieved with BER≈0.1/L, which, for the maximal block length in LTE (*L* = 6144 bits), amounts *BER* ≈ 1.63·10^−5^ and determines the near-optimal “operating point” of (31) to be at $E_{b}/N_{0}\approx 9.3$ dB, whereas the absolute upper-bound *BLER* = 1 is reached with *BER* ≈ 1.63·10^−4^ at $E_{b}/N_{0}=8.1$ dB already, i.e., with just as little as 1.2 dB SNR degradation between the target BLER value and the outage-related one.

Moreover, considering (35), this threshold effect of the LTE physical layer performance, i.e., such a thin margin between the optimal and the outage-state BLER, implies that, for any higher-order RV*_i_*, which has reached the target performance $BLER_{\mathrm{RV}i}\approx 10^{-1}$ with the coding gain $G_{\mathrm{RV}i/0}>1.2$ dB, the RV_0_ was likely with the outage-state performance: $BLER_{\mathrm{RV}0}\approx 1$, as various physical channel impairments (expressed as the AWGN-equivalent abstracts) easily overcome the 1.2 dB margin and produce many erroneous (especially large) blocks.

This finally implies that, for our CP length optimization model, we can justifiably adopt $\Delta BLER_{\mathrm{RV}i/0}\approx 10;i=1,2,3$. 

Moreover, let us consider which value for the BLER reduction $\Delta BLER_{\mathrm{RV}0/\mathrm{UNC}}$ between the RV_0_ and the uncoded block transmission, to adopt in (29) and (30).

**Figure 4:**
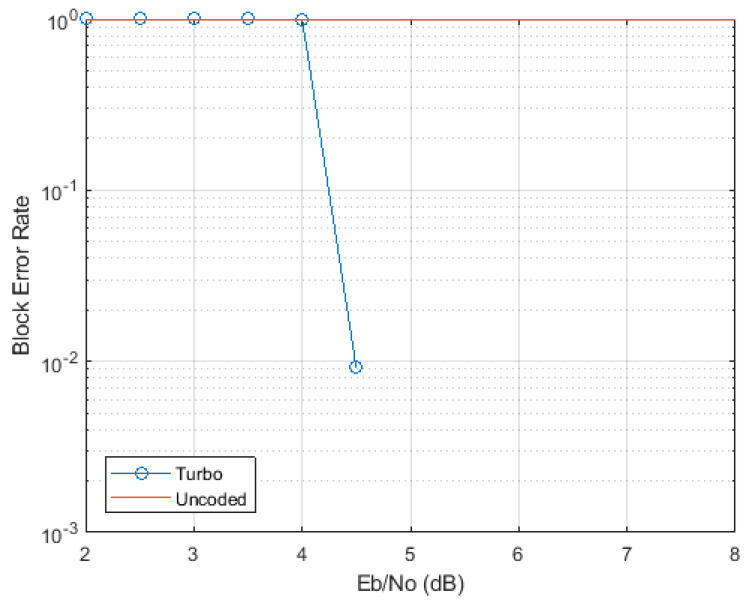
AWGN uncoded vs. turbo-coded BLER for 16 QAM with *L* = 6144.

With this regard, we verified [29] that, with *L* taking the maximal value of 6144 bits, the steepness of the $BLER_{\mathrm{UNC}}$ curves is as much as of those for $BLER_{\mathrm{RV}i}$ in Figure 3, so that again BLER degradation from the projected value of 10^−1^ to the “saturating” value 1 occurs with just a fraction of dB of *E*_b_/*N*_0_ degradation, as it is shown for 16 QAM in the illustrative Figure 4.

This confirms that we can adopt $\Delta BLER_{\mathrm{RV}0/\mathrm{UNC}}=10$, as well.

Thus, to summarize, we can adopt uniform *BLER* reductions of 10 for all RVs, as well as for the uncoded block: $\Delta BLER_{\mathrm{RV}0/\mathrm{UNC}}=\Delta BLER_{\mathrm{RV}i}=10;i=1,2,3$.

### 3.2. Analysis of Numerical Results

Finally, we verify the CP optimization model for the three LTE modulation types and the rms delay spreads of up to 400 ns, attributed to the propagation environments ranging from indoor to typical urban [22], where the latter is included since, nowadays, its median rms delay spread is significantly reduced, as is pointed out in Section 2.2.

Accordingly, by the above-described single-cluster exponential average power delay profile, 1,000,000 instantaneous profiles were generated and subjected to the CP “window” providing the CP-residual rms delay spread values for the optimal CP length estimation (29). In the corresponding Monte Carlo (MC) simulations, bit-error occurrences within erroneous codeblocks were modelled as samples without replacement (Section 2.2), downscaling the incidence of higher-order retransmissions by 10, until the final one is reached.

Thus, based on (30), the CP-related duration ${\bar{L}}_{\mathrm{CB}}\left(\tau_{\mathrm{CP}}^{}\right)\cdot \tau_{\mathrm{CP}}$ of the average gross codeblock was graphed for each selected modulation type and delay spread value, to identify the curve minimum and then checked whether it matched the corresponding optimal $\tau_{\mathrm{CP}}$ value, estimated by (29). 

**Figure 5:**
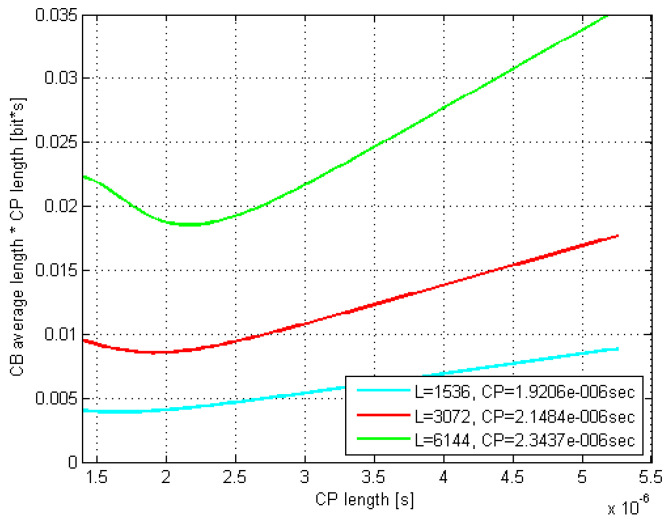
Estimated CB average gross length vs. CP length; DS = 200; 16 QAM.

**Figure 6:**
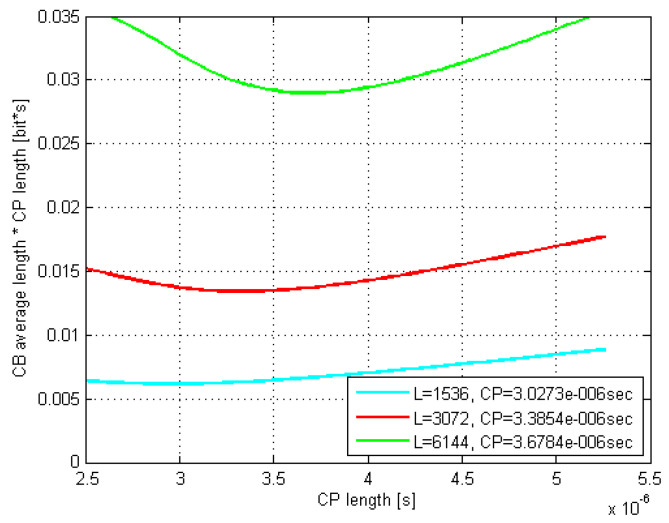
Estimated CB average gross length vs. CP length; DS = 300 ns; 16 QAM.

The according exemplar 16 QAM plots in Figure 5 and Figure 6 are related to the rms delay spreads of 200 and 300 ns. As it can be seen, the curves minima closely match the estimated optimal $\tau_{\mathrm{CP}}$ values.

**Figure 7:**
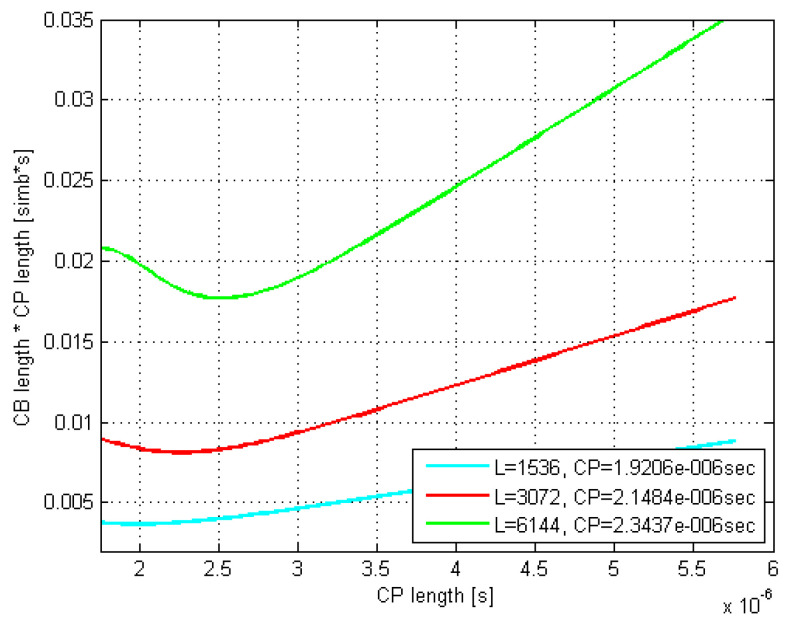
MC-simulated CB average gross length vs. CP length; DS = 200; 16 QAM.

**Figure 8:**
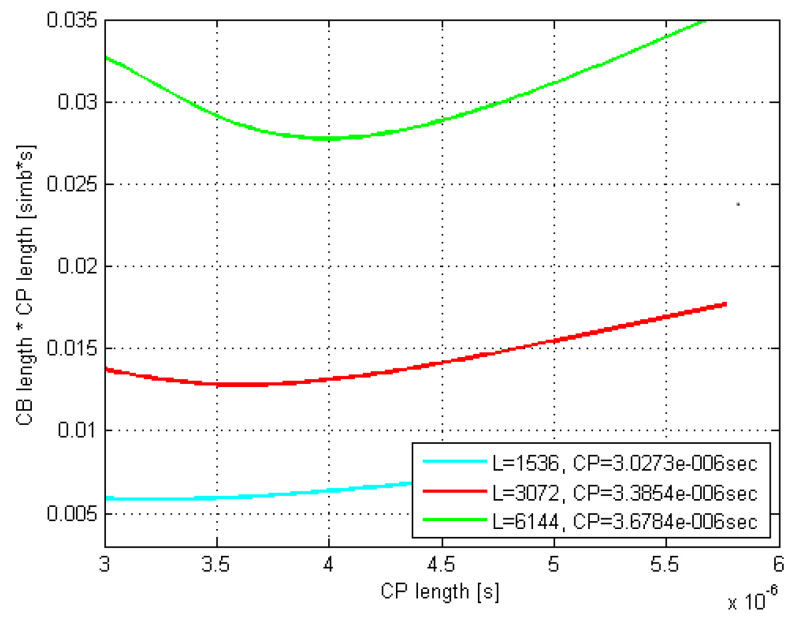
MC-simulated CB average gross length vs. CP length; DS=300 ns; 16 QAM.

Moreover, the optimal CP length as well as ${\bar{L}}_{\mathrm{CB}}\left(\tau_{\mathrm{CP}}\right)\cdot \tau_{\mathrm{CP}}$ values coming out of the related Monte Carlo (MC) simulations were found to match very well to their counterparts estimated by (29) and (30). Thus, e.g., by comparing Figure 7 and Figure 8 with Figure 5 and Figure 6, respectively, it is evident that the minima of the corresponding curves exhibit just a minor offset one to each other.

Furthermore, it is evident that as the codeblock length *L* increases up to 6144 bits, both the optimal $\tau_{\mathrm{CP}}$ and its corresponding ${\bar{L}}_{\mathrm{CB}}\left(\tau_{\mathrm{CP}}^{}\right)\cdot \tau_{\mathrm{CP}}$ increase, whereas the curves’ minima shift to the right and move up, respectively. Moreover, this curves’ dispersion with *L* gets even more pronounced as the rms delay spread increases, which is in accordance with (29).

Thus, the optimal CP length with the maximal codeblock length of 6144 bits (providing least CP-optimization benefit) and the rms delay spreads of 100, 200, 300, and 400 ns, was found to be equal to 1.11, 2.41, 3.78, and 5.03 μs for 4 QAM modulation type, 1.07, 2.34, 3.68, and 4.91 μs for 16 QAM, and 1.04, 2.25, 3.58, and 4.83 μs for 64 QAM, respectively, whereas the normal CP of 4.69 µs was reached with the rms delay spread values of 3.70, 3.79, and 3.87 ns, for the respective modulation types. 

**Figure 9:**
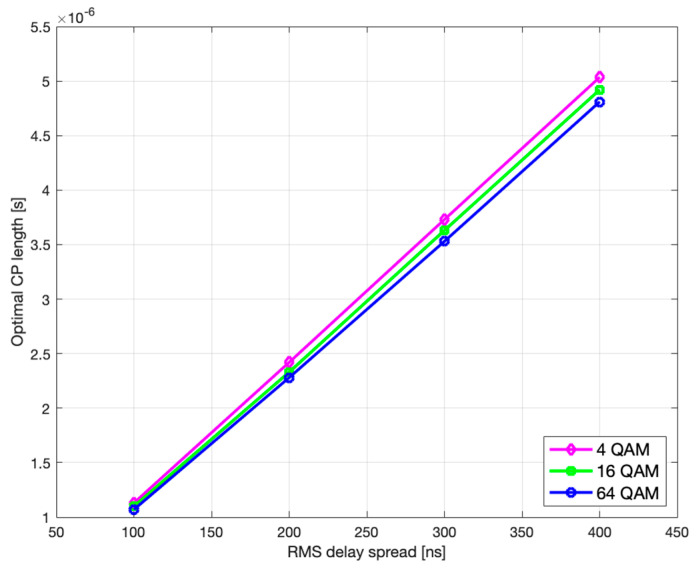
Optimal CP length vs. rms delay spread.

**Figure 10:**
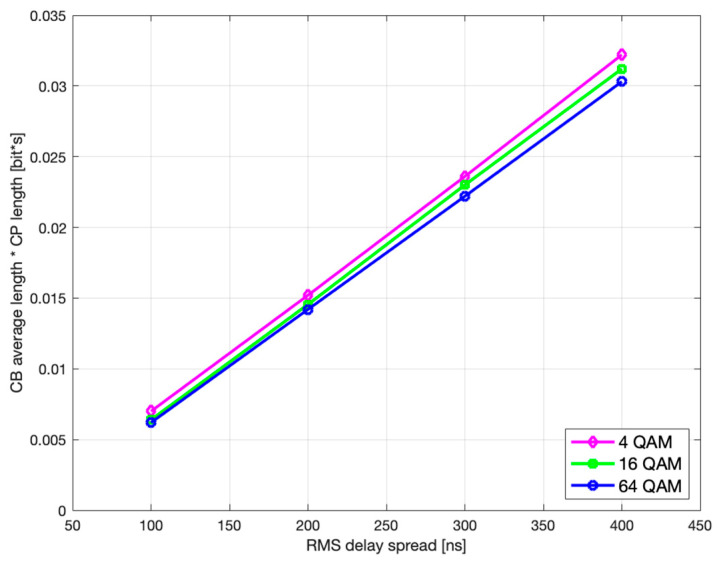
CB average gross length vs. rms delay spread.

Finally, if we observe the rms delay spread values between Figure 5 and Figure 6 (or Figure 7 and Figure 8), it is noticeable that for the same modulation type, increasing the rms delay spread is expectedly tracked by increasing both the optimal $\tau_{\mathrm{CP}}$ and the corresponding CP-related duration ${\bar{L}}_{\mathrm{CB}}\left(\tau_{\mathrm{CP}}^{}\right)\cdot \tau_{\mathrm{CP}}$ of the codeblock average gross length. To what extent are the increments of the latter two related to the rms delay spread is of particular interest here and is therefore accordingly presented in Figure 9 and Figure 10, for *L* = 6144. 

As it can be seen, both the optimal $\tau_{\mathrm{CP}}$ and ${\bar{L}}_{\mathrm{CB}}\left(\tau_{\mathrm{CP}}\right)\cdot \tau_{\mathrm{CP}}$ monotonically increase with rms delay spread.

**Figure 11:**
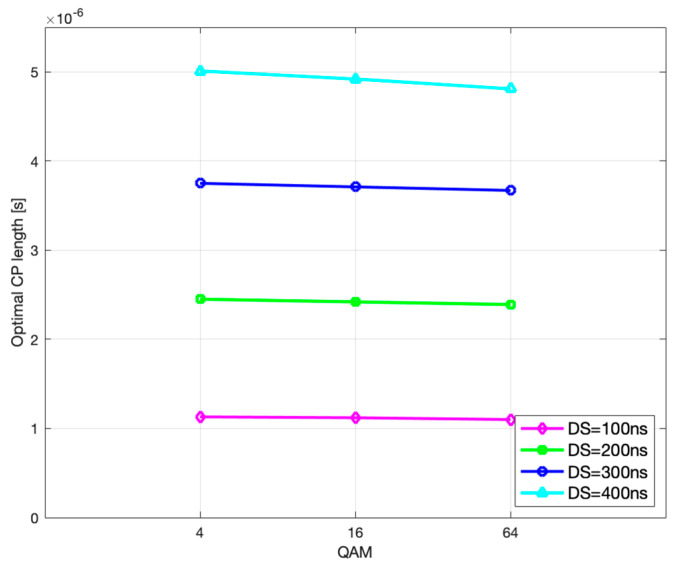
Optimal CP length vs. modulation order.

**Figure 12:**
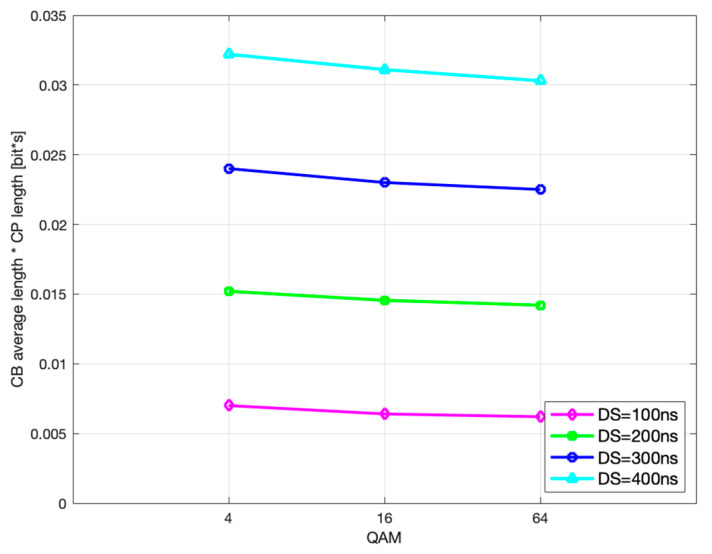
CB average gross length vs. modulation order.

Likewise, further analysis shows that the optimal CP length and the corresponding CP-weighted duration of codeblock average gross length slowly decrease with higher modulation order, as seen in Figure 11 and Figure 12, which is in accordance with (29) and (30), respectively.

Moreover, coming out of the motivation to find the optimal CP length value and compare it to the (suspectedly oversized) normal one of 4.69 μs, the key indicator to quantify the potential benefit is the achievable efficiency gain, i.e., the relative CP-weighted average codeblock length reduction that we define here as:

(36) $$\eta_{G}=\frac{{\bar{L}}_{\mathrm{CB}}\left(4.69\right)\cdot 4.69-{\bar{L}}_{\mathrm{CB}}\left(\tau_{\mathrm{CP}}^{}\right)\cdot \tau_{\mathrm{CP}}^{}}{{\bar{L}}_{\mathrm{CB}}\left(4.69\right)\cdot 4.69}$$

The results of applying (36) onto the obtained CP-weighted codeblock average gross length values, such as the graphically presented ones in Figure 5 and Figure 6 for the case of 16 QAM are given in Table 2, Table 3 and Table 4 for all three modulation types, with chosen rms delay spreads and codeblock lengths.

As it can be seen, particularly with the least-beneficial (maximal) codeblock length of 6144 bits and the rms delay spreads of 100, 200, 300, and 400 ns, the optimization of CP length provides 74.9%, 46.7%, 17.4%, and −19.2% efficiency gain with regard to the normal CP value for 4 QAM modulation type, whereas 76.3%, 47.7%, 19.1%, and −18.5% are the outcomes for 16 QAM, and 77.0%, 49.2%, 21.8%, and −17.6% for 64 QAM, respectively.

Finally, as the CP length optimization model equally applies to testing the standard CP length values of all 5G NR numerologies, just as it does for the LTE (i.e., numerology 0) normal CP, this implies that the above-cited relative values (percentages) also apply for all numerologies, while the absolute values (expressed in μs) need to be properly downscaled by 2 for each next higher numerology.

Thus, to summarize, the above numerical results validate the developed analytical model for assessing adequacy of the industry-standard 4G/5G CP length value(s), clearly indicating that these are significantly oversized.

## 4. Conclusions

A novel and comprehensive crosslayer analytical model is developed to assess appropriateness of the standard CP length value(s), adopted in LTE and 5G NR specifications.

While all other so far reported investigations and qualifications of the industry-standard CP length have been based on the PHY layer alone, the optimal CP length is derived here in such a way as to minimize the effective average codeblock length determined by trade-off between the two mutually complementary mechanisms: reducing the CP length itself at PHY layer and the consequent rising incidence of codeblock repetitions due to error recovery through the layers above PHY—MAC and RLC, in particular.

In this regard, we simplified the analysis by introducing several analytically and numerically justified approximations, so easing the interpretation of the finally obtained expressions.

Thereby, for given protective coding parameters, the optimal CP length is found to be determined by the rms delay spread of the channel power delay profile part exceeding the CP span.

Concretely, with rms delay spreads ranging from 100 to 300 ns, the optimal CP length values are found to significantly reduce the CP-weighted codeblock average gross length with respect to its values achieved with the LTE (i.e., 5G NR numerology 0) normal CP of 4.69 μs, specifically: from 74.9% to 17.4% for the 4 QAM modulation type, from 76.3% to 19.1% for 16 QAM, and from 77.0% to 21.8% for 64 QAM, respectively, whereas the negative reductions (i.e., effective growths) of −19.2%, −18.5%, and −17.6%, found for the rms delay spread of 400 ns, indicate that the standard (normal) CP length of 4.69 µs is close to optimal for that extent of time dispersion, as it is reached with the rms delay spread values of 370, 379, and 387 ns, for the respective modulation types.

These numerical results validate the developed analytical CP length model and undoubtedly unveil that the industry-standard CP lengths are significantly oversized, unnecessarily reducing the net throughput in propagation environments ranging from indoor to typical urban.

This work was aimed to discover, verify and quantify the potential for reducing the CP overhead and so pave the way to according R&D and field tests taking into account design and deployment issues as well, and using sophisticated hardware and industry-standard software simulation tools.

## Figures and Tables

**Table 1:** 5G NR numerology.

Parameter/Numerology	Subcarrier Spacing (kHz)	OFDM Symbol Length (μs)	CP Length (μs)
0	15	66.67	4.69
1	30	33.33	2.34
2	60	16.67	1.17
3	120	8.33	0.57
4	140	4.17	0.29

**Table 2:** CP-weighted average codeblock length reduction for 4 QAM.

Rms Delay Spread	Codeblock Length		
$\sqrt{\overset{-}{\tau_{i}^{2}}}$	*L* = 1536	*L* = 3072	*L* = 6144
100 ns	79.3%	77.7%	74.9%
200 ns	56.1%	51.1%	46.7%
300 ns	29.6%	22.5%	17.4%
400 ns	3.4%	−17.0%	−19.2%

**Table 3:** CP-weighted average codeblock length reduction for 16 QAM.

Rms Delay Spread	Codeblock Length		
$\sqrt{\overset{-}{\tau_{i}^{2}}}$	*L* = 1536	*L* = 3072	*L* = 6144
100 ns	80.6%	77.9%	76.3%
200 ns	57.0%	53.2%	47.7%
300 ns	31.3%	26.0%	19.1%
400 ns	7.6%	−16.1%	−18.5%

**Table 4:** CP-weighted average codeblock length reduction for 64 QAM.

Rms Delay Spread	Codeblock Length		
$\sqrt{\overset{-}{\tau_{i}^{2}}}$	*L* = 1536	*L* = 3072	*L* = 6144
100 ns	81.4%	79.2%	77.0%
200 ns	59.1%	53.5%	49.2%
300 ns	34.7%	27.1%	21.8%
400 ns	9.6%	−14.9%	−17.6%

## Data Availability

Not applicable.
